# Neural Networks With Motivation

**DOI:** 10.3389/fnsys.2020.609316

**Published:** 2021-01-11

**Authors:** Sergey A. Shuvaev, Ngoc B. Tran, Marcus Stephenson-Jones, Bo Li, Alexei A. Koulakov

**Affiliations:** ^1^Cold Spring Harbor Laboratory, Cold Spring Harbor, NY, United States; ^2^Sainsbury Wellcome Centre, University College London, London, United Kingdom

**Keywords:** machine learning, motivational salience, reinforcement learning, artificial intelligence, addiction, hierarchical reinforcement learning

## Abstract

Animals rely on internal motivational states to make decisions. The role of motivational salience in decision making is in early stages of mathematical understanding. Here, we propose a reinforcement learning framework that relies on neural networks to learn optimal ongoing behavior for dynamically changing motivation values. First, we show that neural networks implementing Q-learning with motivational salience can navigate in environment with dynamic rewards without adjustments in synaptic strengths when the needs of an agent shift. In this setting, our networks may display elements of addictive behaviors. Second, we use a similar framework in hierarchical manager-agent system to implement a reinforcement learning algorithm with motivation that both infers motivational states and behaves. Finally, we show that, when trained in the Pavlovian conditioning setting, the responses of the neurons in our model resemble previously published neuronal recordings in the ventral pallidum, a basal ganglia structure involved in motivated behaviors. We conclude that motivation allows Q-learning networks to quickly adapt their behavior to conditions when expected reward is modulated by agent’s dynamic needs. Our approach addresses the algorithmic rationale of motivation and makes a step toward better interpretability of behavioral data via inference of motivational dynamics in the brain.

## Introduction

*Motivational salience* is a cognitive process that motivates, or propels, an individual’s behavior toward or away from a particular object, event, or outcome (Zhang et al., 2009). Such process describes an *a priori* defined “wanting” of an outcome. It regulates behaviors toward particular goals, adjusts the amounts of time and energy that an individual is willing to expend in pursuit of each desired outcome, and sets the acceptable levels of related risk (Zhang et al., 2009; Berridge, 2012). Motivational salience, or, as we will call it here for brevity, motivation, describes animals’ *a priori* desire or aversion to receive a particular outcome, which should be contrasted with liking or disliking of an outcome that is experienced *a posteriori*. Mathematically, motivation can be viewed as a subjective modulation of the expected value of reward, determined before the reward is received.

Behavior-based models of motivation emerged as a part of the broader effort to understand reward-guided behaviors in humans and other animals [reviewed by Miller (2008)]. Motivational levels in these models were described as the subjects’ *drives* toward certain outcomes (e.g., appetite and thirst). To estimate the relative dynamics of different drives (not observable in experiment) psychologists offered human or animal subjects to approach/avoid different combinations of stimuli and titrated responses based on the valency/strength of these inputs (Sears and Hovland, 1941; Miller et al., 1943). Such “conflict” experiments resulted in detailed models of motivational dynamics. Motivational drives can therefore be viewed as temporarily varying representations of motivational salience.

Neuronal correlates of motivation-related variables were discovered in the *ventral pallidum* (VP). VP is a part of the basal ganglia that receives the inputs from a number of mesocorticolimbic areas (Kelley et al., 1982; Reep and Winans, 1982; Fuller et al., 1987; Grove, 1988; Martinez-Murillo et al., 1988; Heimer et al., 1991; Maslowski-Cobuzzi and Napier, 1994; Maurice et al., 1997; Berridge, 2012). As the major output of the ventral basal ganglia (Saper and Loewy, 1980; Heimer et al., 1987; Mogenson and Yang, 1991; Leung and Balleine, 2013), it sends substantial projections to the lateral habenula (LHb), dorsal and medial raphe nuclei (DR/MR), ventral tegmental area (VTA), substantia nigra pars compacta and pars reticulata (SNc and SNr), and mediodorsal thalamus (Haber and Knutson, 2010; Richard et al., 2016). Thus, the VP is a hub linking areas involved in reward processing with motor output regions, and is anatomically poised to mediate motivated behaviors. Indeed, lesions in the VP induce aphagia and adipsia, the lack of motivation to eat and drink, respectively (Morgane, 1961; Stellar et al., 1979; Humphries and Prescott, 2010), and anhedonia, an inability to feel pleasure (Berridge, 1996). An intact VP is also necessary for drug seeking behaviors (McFarland and Kalivas, 2001; Harvey et al., 2002; Miller et al., 2006; Vijayaraghavan et al., 2008) and for active avoidance and aversive learning (Ishihara et al., 1991; Page et al., 1991; Root et al., 2013). Human brain imaging studies indicate that the VP activities correlate with motivational vigor (Pessiglione et al., 2007; Root, 2013; Singh-Bains et al., 2016). *In vivo* single unit recording studies in rodents and monkeys indicate that the VP neuron firing correlates with motivational salience (Berridge and Schulkin, 1989; Tindell et al., 2004; Smith and Berridge, 2007; Tachibana and Hikosaka, 2012; Jiang et al., 2015; Richard et al., 2016). In the experiments in which sodium starvation was introduced in rats, the responses of the VP neurons to the conditioned stimulus (CS) associated with normally aversive sodium stimulus have changed to match those to the CS associated with normally attractive sucrose (Berridge, 2012). These observations suggest that the VP is critically involved in “positive motivation,” including the “liking” (the pleasurable impact of reward consumption) and the “wanting” (the attractiveness of a stimuli or incentive salience) aspects of behaviors (Berridge and Schulkin, 1989). It may also be involved in “negative motivation”, the drive to avoid aversive stimuli. In this study, we investigated the circuit mechanism of representation of motivational information in VP networks.

Computational models for motivated behaviors are best represented by *reinforcement learning* (RL) models. RL is the area of machine learning and artificial intelligence that deals with the strategies that rational agents can employ while navigating in an environment to maximize future rewards (Sutton and Barto, 1998; Zhang et al., 2009). As such, RL models are successful in predicting and explaining adaptive choice behaviors in both human and animals, and have been successful in predicting the causal changes in neuronal responses (Schultz et al., 1997; Schultz, 1998; Dayan and Abbott, 2001; Lee et al., 2012). The underlying RL theory has been widely adapted as a framework for both interpreting experimental data and designing new experiments (Schultz, 2007; Lee et al., 2012). Specifically, it is successful in explaining how the brain adjusts the estimates of future rewards and updates these expectations based on experience (Schultz et al., 1997; Schultz, 1998; Dayan and Abbott, 2001; Lee et al., 2012).

Motivation has been approached in RL from multiple angles. In the research on *intrinsic motivation* the agents were additionally rewarded for exercising “curiosity” to try new strategies useful for prospective goals (Chentanez et al., 2005; Singh et al., 2010; Kulkarni et al., 2016). In *multi-objective RL* (MORL), motivations affected the available actions favoring particular behaviors to prioritize certain objectives [reviewed in Liu et al. (2014)]. In both intrinsic motivation and MORL, the concepts of motivation were introduced to achieve certain computational flexibility with no focus on building a plausible model of the human/animal decision-making.

RL models are mostly concerned with the learning aspect of behavior. However, fluctuations in physiological states can profoundly affect behavior. Recent suggestions include using time-varying multiobjective reward functions in biological context (Koulakov, 2018; Palm and Schwenker, 2019). Modeling such factors is thus an important goal in computational neuroscience and is in the early stages of mathematical description (Berridge, 2012; Berridge and Robinson, 2016). In this study, we develop a computational network model of motivational salience in the context of RL. Since RL relies on future rewards to generate behavior, and these rewards are modulated by motivational states, complete understanding of complex behavioral choices is impossible without incorporating motivation. We compare the results of our model to the previously published mouse data obtained in the classical conditioning paradigm (Stephenson-Jones et al., 2020), in which recordings from the VP neurons are available. We show that our motivated RL model both learns to correctly predict motivation-dependent rewards/punishment and generates neural responses consistent with the responses of the VP neurons. In particular, we show that, similarly to real neurons, RL neural networks contain two oppositely-tuned populations of neurons responsive to rewards and punishment. In the model, these two populations form a recurrent network that helps maintain motivation-dependent variables when inputs are missing. Our RL-based model is both consistent with previously published experimental data and suggests a hypothesis for the structure of connectivity in the VP networks. We show that networks with motivation can adapt their behavior to changes in reward functions without relearning network weights and can do so without prespecified goals. We demonstrate how our network model can form the basis of the hierarchical RL system. Overall, we argue that neural networks implementing motivational salience in the brain may enable compact representation of dynamic behaviors accommodating to the shifts in the needs of agents.

## Results

### Berridge’s Model of Motivation in Reinforcement Learning Framework

The goal of this work is to analyze the mathematical/algorithmic implications of motivational salience, and to explore its neuronal substrates. Motivation can be defined as a need-dependent modulation of the expected subjective value of an anticipated reward, depending on an animal’s intrinsic conditions (Zhang et al., 2009). Thus, rats, which are normally repelled by high levels of salt in their food, may become attracted to a salt-containing solution following the reduced-sodium diet (Berridge, 2012). To model this observation, the subjective value of reward $\tilde{r}$ may be considered as not being absolute, but rather modulated by an internal variable reflecting the level of motivation (Berridge and Schulkin, 1989), which we will call here μ. The subjective value of the reward $\tilde{r}$ based on the motivation μ and the physical reward magnitude *r* can be expressed by equation $\tilde{r}=\tilde{r}\,\left(r,\mu \right)$.

In the simplest example, the subjective value of a reward associated with salt may be given by $\tilde{r}=\mu \cdot r$. Baseline motivation toward salt can be defined by μ = −1, leading to the negative subjective reward value of $\tilde{r}=-r\leq 0$. Thus, normally, the presence of salt in the rats’ diet is undesired. In the reduced-sodium condition, the motivation is changed to μ = + 1 leading to the positive subjective reward value of $\tilde{r}=+r\geq 0$. Thus salt-containing diet becomes attractive. In reality, the function $\tilde{r}\,\left(\ldots \right)$ defining the impact of motivation on a subjective value of reward may be more complex (Zhang et al., 2009) including the dependence on multiple factors described by a motivation vector $\vec{\mu }$. Individual components of this vector describe various needs experienced by the organism, such as thirst (e.g., μ_*1*_), hunger (μ_*2*_), etc. The scalar subjective value $\tilde{r}$ of the reward includes all physical rewards and relies on vectors of physical reward amounts $\vec{r}$ and an agent’s motivations $\vec{\mu }$:

(1) $$\tilde{r}=\tilde{r}\,\left(\vec{r},\vec{\mu }\right)$$

In this work, we explore how motivation vector $\vec{\mu }$ affects behaviors through modulating physical reward values $\vec{r}$. We also investigate brain circuits that may implement such computations.

Our model is based on the Q-learning (Watkins and Dayan, 1992) – a fundamental RL algorithm for choosing optimal strategies to maximize rewards (Sutton and Barto, 1998). In the Q-learning, an agent estimates the sum of future rewards (Q-function), which depends on the agent’s current *state* ${\vec{s}}_{t}$, such as position in the environment, and potential choices of *action a*_*t*_. The agent picks an action *a*_*t*_ to maximize the Q-function. If motivation is taken into account, the *expected subjective* values of the reward ${\tilde{r}}_{t}$ change with the motivational vector ${\vec{\mu }}_{t}$, and the Q-function depends on three parameters:

(2) $$Q\,\left({\vec{s}}_{t},a_{t},{\vec{\mu }}_{t}\right)=\text{E}\,\left[\sum_{\tau =0}^{\infty }\tilde{r}\,\left({\vec{s}}_{t+\tau },{\vec{\mu }}_{t+\tau }|a_{t}\right)\,\gamma^{\tau }\right]$$

Here 0 < γ≤1 is the discounting factor that balances the preference between short-term and long-term rewards. To learn motivation-dependent Q-functions, agents in our model use the Time Difference (TD) method (Sutton and Barto, 1998) as follows. When perfectly learned, a Q-function satisfies the recursive relationship known as the Bellman equation (Sutton and Barto, 1998):

(3) $$Q\,\left({\vec{s}}_{t},a_{t},{\vec{\mu }}_{t}\right)=\tilde{r}\,\left({\vec{s}}_{t},{\vec{\mu }}_{t}\right)+\gamma \,\max_{a_{t+1}}Q\,\left({\vec{s}}_{t+1},a_{t+1},{\vec{\mu }}_{t+1}\right)$$

For incompletely learned Q-functions, the equation above is not exact. To update the values of the Q-function, agents compute the discrepancy between the sides of the Bellman equation, termed the TD error:

(4) $$\delta =\tilde{r}\,\left({\vec{s}}_{t},{\vec{\mu }}_{t}\right)+\gamma \,\max_{a_{t+1}}Q\,\left({\vec{s}}_{t+1},a_{t+1},{\vec{\mu }}_{t+1}\right)-Q\,\left({\vec{s}}_{t},a_{t},{\vec{\mu }}_{t}\right)$$

Learning motivation-dependent Q-functions may become computationally costly: in addition to sampling possible states ${\vec{s}}_{t}$ and actions *a*_*t*_, agents with motivation have to compute the Q-function values for a variety of motivations ${\vec{\mu }}_{t}$. To avoid separate learning of the Q-function values for each possible combination of the input parameters, we approximate the Q-functions with artificial neural networks and use the TD errors as a training signal.

In our model, we adjust the conventional TD learning approaches to the tasks where future rewards are modulated by motivation. The new set of variables $\vec{\mu }$ reflecting various components of motivation evolves in accordance with its own rules. Below we use our model to evaluate the behavioral implications of motivational modulation of rewards, and to infer motivational state/dynamics of behaving agents. We further show consistency of our model with neuronal activations recorded in the ventral pallidum (VP) in our previous work (Stephenson-Jones et al., 2020).

### Four Demands Task

**FIGURE 1:**
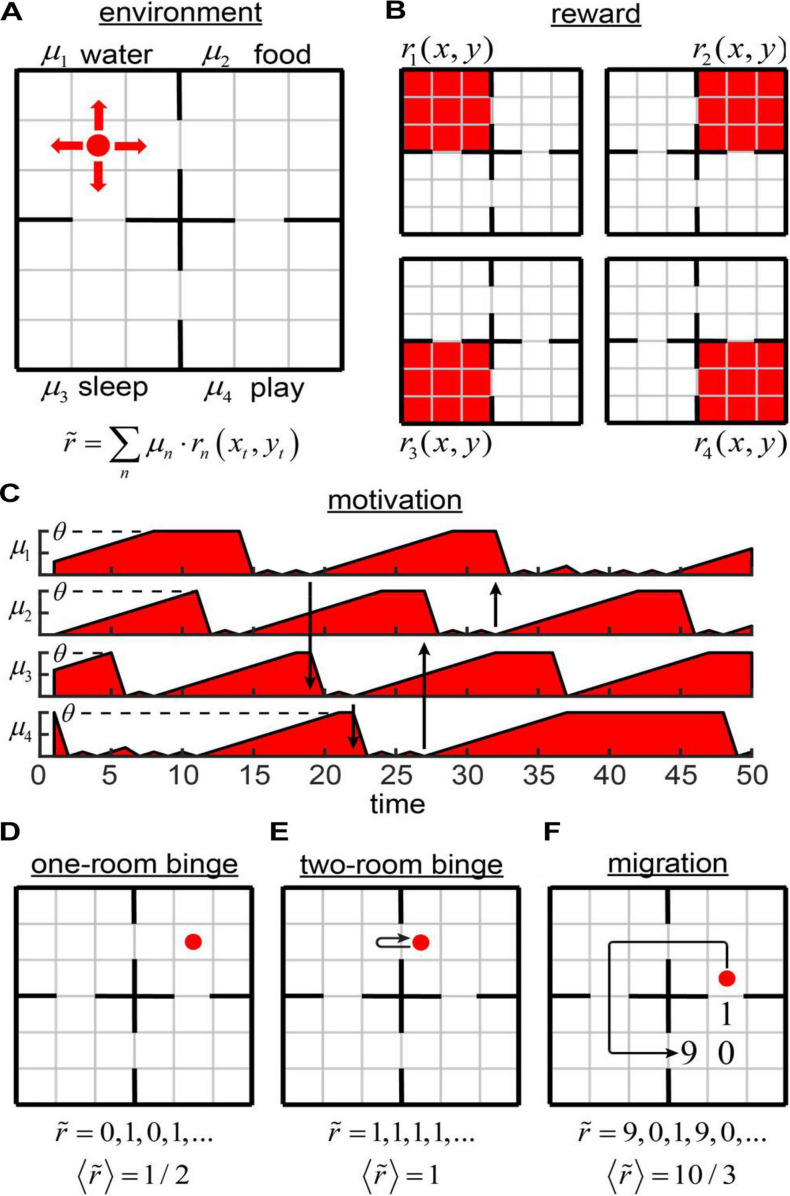
The Four Demands task. **(A)** An agent inhabits a 6 × 6 environment separated into four rooms. Each room is associated with its own reward and motivation (water, food, sleep, and play). **(B)** Components of physical reward values (color coded: red = 1, white = 0). The subjective reward value is a scalar product between the motivation vector and the physical reward value vector as illustrated. **(C)** Possible components of the 4D motivation vector as functions of time. Arrows indicate some of the transitions between the rooms. When the agent enters a room, its motivation is reset to zero. When the agent does not receive a non-zero perceived reward available in the room, the motivation increases by 1 at each time step until saturation at **θ**. **(D–F)** Potential strategies in our model: one-room binge **(D)**, two-room binge **(E)**, and migration **(F)**.

To introduce networks with motivation, we considered the example of Four Demands task (Figure 1). An agent navigates in a 6 × 6 square grid world separated into four 3 × 3 subdivisions (rooms) (Figure 1A). The environment was inspired by the work of Sutton et al. (1999); however, the task is different, as described below.

In each room, the agent receives one and only one type of reward *r*_*n*_(*x*_*t*_,*y*_*t*_), where *n* = 1..4 (Figure 1B). The value of each reward component equals to 1 in a corresponding room (Figure 1B, red color), and to 0 elsewhere (Figure 1B, white color). These rewards can be viewed as four different resources, such as water, food; an ability to sleep, and play a game. Motivation is described in this system by a 4D vector $\vec{\mu }$ defining affinity of the agent for each of these resources. When the agent enters a room number *n*, the agent receives the reward defined by its subjective value $\tilde{r}=\left(\vec{\mu }\cdot \vec{r}\right)=\mu_{n}$ and the corresponding component of the motivation vector is reset to zero μ_*n*_→0 (Figure 1C) assuming that the agent is sated w.r.t the current reward type, and that such satiation happens fast compared to building up motivation toward a reward type. At the same time, motivations in the other three rooms are increased by one, i.e., μ_*others*_→μ_*others*_ + 1, which reflects additional “wanting” of the resources induced by the “growing appetite.” After a prolonged period of building up appetite, the motivation toward a resource saturates at a fixed maximum value of θ, which becomes a parameter of this model. Behavior of the agent is determined by this parameter. Such a model is consistent with previous studies suggesting that, in the absence of stimuli, corresponding drives increase at idiosyncratic paces until eventual saturation (Miller, 2008).

What are the potential behaviors of the agent? Assume, for simplicity, that the maximum allowed motivation θ is large and does not influence our results. If the agent always stays in the same room (one-room binge strategy, Figure 1D), the subjective values of the rewards received by the agent consist of a sequence of zeros and ones, i.e., 0, 1, 0, 1, … This is because, in our model, after the motivation is set to zero, it is increased by one on the next time step. The average subjective value of the reward rate $\left\langle \tilde{r}\right\rangle =E\,\left[\tilde{r}\right]$ corresponding to this strategy is therefore ${\left\langle \tilde{r}\right\rangle }_{o\,n\,e-r\,o\,o\,m-b\,i\,n\,g\,e}=1/2$. The average subjective value of the reward rate can be increased, if the agent jumps from room to room at each time step. This is what we call a two-room binge strategy (Figure 1E). In this case, the sequence of subjective values of the rewards received by the agent is described by the sequence of ones, and the average subjective reward rate is ${\left\langle \tilde{r}\right\rangle }_{t\,w\,o-r\,o\,o\,m-b\,i\,n\,g\,e}=1$. Two-room binging therefore outperforms the one-room binge strategy. Finally, the agent can migrate by moving in a cycle through all four rooms (Figure 1F). In this case, the agent spends three steps in each room and the overall period of migration is 12 steps. During three steps spent in a single room, an agent receives the rewards subjectively valued as 9 (the agent left this room nine steps ago), 0, and 1. The average subjective reward rate per iteration is ${\left\langle \tilde{r}\right\rangle }_{m\,i\,g\,r\,a\,t\,i\,o\,n}=10/3$. Thus, migration strategy is more beneficial for the agent than both of the binging strategies. Migration, however, is affected by the maximum allowed motivation value θ. When θ < 9, the benefits of migration strategy are reduced. For θ = 1, for example, migration yields the subjective reward rate of just ${{\left\langle \tilde{r}\right\rangle }_{m\,i\,g\,r\,a\,t\,i\,o\,n}|}_{\theta =1}=2/3$, which is below the yield of the two-room binging. Thus, our model is expected to display various behaviors depending on θ.

**FIGURE 2:**
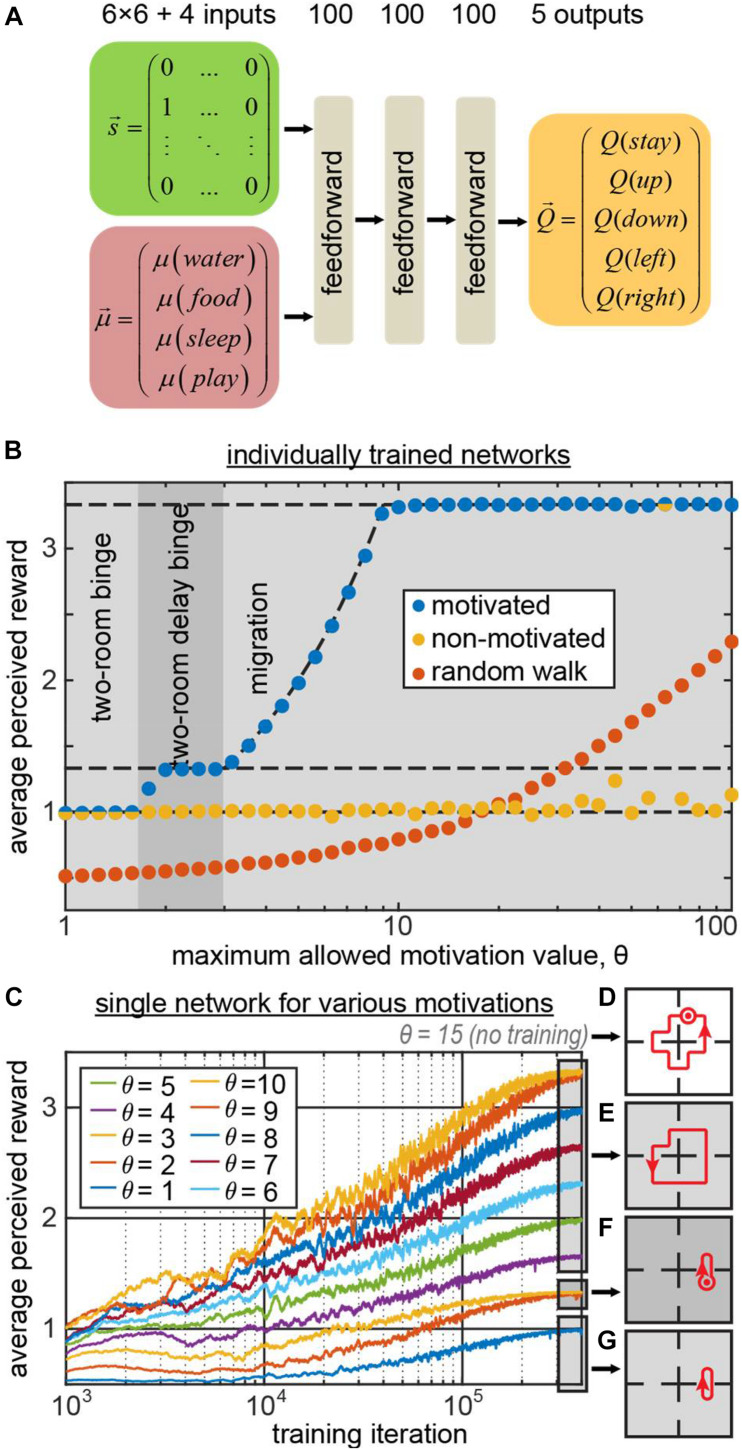
**(A)** The architecture of the 3-layer fully connected network computing the Q-function *$Q\,\left(a|\vec{s},\vec{\mu }\right)$*. **(B)** The average subjective reward rate received by the network trained with maximum allowed motivation value θ (blue circles – motivation is provided as an input to the network; yellow – motivation affects the reward as usual, but is not provided to the network; orange – random walk). The regions of θ corresponding to different optimal strategies, learned by the motivated agent, are shown by different gray areas in the plot. These areas represent the phase diagram of the optimal behaviors displayed by the motivated agent. The dashed lines indicate the expected subjective reward values associated with these strategies: top – migration; middle – two-room delay binge; bottom – two-room binge. For small/large values of θ, the motivated network displays two-room binge/migration behaviors, respectively. Under the same conditions, the non-motivated network mostly displays two-room binge behavior. **(C)** A single network trained in minibatches for various values of θ, which in this case was a separate, 41st input to the network. Different curves correspond to different values of θ. For θ = 4..10/θ = 2,3/θ = 1, the model exhibits the migration/two-room delay binge/two-room binge strategies depicted in **(E–G)** respectively (dot with the circle denotes staying at the same location one extra step). **(D)** For the novel input θ = 15 that was not used in training, the model displays a new strategy, delayed migration.

We trained a simple feedforward neural network (Figure 2A) to generate behaviors using the state vector and the 4D vector of motivations as inputs. The network computed the Q-values for five possible actions (up, down, left, right, stay) using TD method and backpropagating the TD error δ (the details are provided in the “Materials and Methods” section). The binary 36D (6 × 6) one-hot state vector represented the agent’s position (“1000…” for the leftmost square in the top row, “0100…” for the second square in the top row, etc.). We trained the network 41 times for different values of the maximum allowed motivation value θ. As expected, the behavior displayed by the network depended on this parameter. The phase diagram of the agent’s behaviors (Figure 2B blue circles) shows that the agent has successfully discovered the migration and two-room binge strategies for high and low values of θ correspondingly (blue circles indicating the agents’ subjective reward rates match the reward rates of corresponding optimal strategies, indicated with the dashed lines in Figure 2B. For intermediate values of θ(1.7 < θ < 3), the network has discovered a delayed two-room binging strategy, in which it spent an extra step in one of the rooms.

Does knowing motivation help learning optimal strategies? To address this question, we performed a similar set of simulations, except the motivation input to the network was replaced with zeros $\left({\vec{\mu }}^{*}=0\right)$. Although the information about the “true” motivation vector $\vec{\mu }$ was not available to the agent, the dynamics of the “true” vector $\vec{\mu }$ was computed in the simulation as described before and affected the subjective reward values $\tilde{r}=\left(\vec{\mu }\cdot \vec{r}\right)\neq \left({\vec{\mu }}^{*}\cdot \vec{r}\right)$. This way, the rewards in this simulation matched the rewards in the previous simulation, corresponding to the same optimal strategies. Although the input to such “non-motivated” networks was sufficient to recover the optimal strategies, in most of the simulations, the agents exercised two-room binging (Figure 2B, yellow circles). The migration strategy, despite being optimal in most simulations, was successfully learned only by a single agent out of 41. Moreover, the performance of the non-motivated networks was worse than that of the random walk (Figure 2B, orange circles) in 13 simulations out of 41, corresponding to large values of θ.

Such relative success of the random walk strategy at the large values of θ can be explained by the fact that eventually random walk brings an agent to each of the rooms. When the maximum allowed motivation θ is large, regardless of how long an agent did not visit a room, the motivation in it continues to increase by one at every time step allowing a random walk agent to eventually harvest a subjectively large reward. At the same time, non-motivated agents learned the two-room binging strategy: it is an easy strategy to learn, as it requires considering only one step ahead, and minimizes the TD error (the network can perfectly predict the reward on the next step). More optimal strategies, such as the migration strategy, require considering many steps ahead thus necessitating an association between current action and reward in distant future, known as the temporal credit assignment problem. In motivated agents, motivation provides explicit cues about the expected subjective reward value dynamics and thus may help solving the temporal credit assignment problem. At the same time, non-motivated agents do not have access to the cues about the subjective reward value dynamics and fall short of building long-range associations between their actions and resulting reward. Therefore, non-motivated agents may only learn the few-step strategies, such as the two-room binging, where they can easily predict the next reward. We therefore conclude that motivation may facilitate learning by providing additional cues for temporal credit assignment in the reward dynamics. Overall, we suggest that motivation is helpful in generating prolonged multi-step behaviors, such as the migration strategy.

In the previously described example, different networks were trained for each value of motivation maximum θ. Can a single network learn all of these strategies within a fixed set of weights, and switch its behaviors when parameter θ changes? To address this question, we trained a single network to behave in trials with different maximum values of motivation, θ. To indicate to the network the current value of θ in each trial, we explicitly provided the network with an additional input, indicating the value of θ (see section “Materials and Methods” for more details). We show (Figure 2C) that the network has indeed learned the optimal policies, corresponding to each motivational schedule (Figures 2E–G). Moreover, it can switch between different behavioral strategies immediately without the need to relearn them, based on the external input. We therefore propose that motivation may constitute a mechanism allowing neural networks to switch between different behaviors, satisfying an agent’s changing requirements, without the need for changes in synaptic strengths.

In previous work, conceptualization of incentive motivational salience stated that the value of cues can be dynamically modulated in novel motivational states without the opportunity for new learning about cues or rewards in that new state (Zhang et al., 2009). To observe whether our network model accounts for this finding, we have evaluated our network (trained for the maximum allowed motivation values of 1–10) with the new maximum motivation value of 15. The network model has not encountered such motivation value during training, and has to extrapolate its knowledge of the environment/motivation dynamics to behave optimally in such setting. We found that the network has executed the new “delayed migration” strategy where it went in circles through all four rooms with an extra “stop” for two iterations in one of the rooms (Figure 2D). Such strategy yielded the average subjective reward value of 47/14 = 3.36 exceeding the yield of the usual “migration” strategy (10/3 = 3.33) described previously. Thus, our deep network model learned to generalize the relationship between motivation and reward, and was able to extrapolate its behavior to a novel motivational context (zero-shot learning) via developing a new strategy – in line with the previous conceptualization of motivational salience.

Overall, we suggest that neural networks with motivation are suited to quickly learn a compact representation of various behaviors and have the flexibility to switch between them quickly when the needs of an agent shift. Such changes in behavior can be accomplished without relearning the synaptic strengths. This feature gives neural networks with motivation an advantage compared to standard RL models which have to relearn behaviors when agent’s needs change.

### Four Demands Network With “Addiction”

**FIGURE 3:**
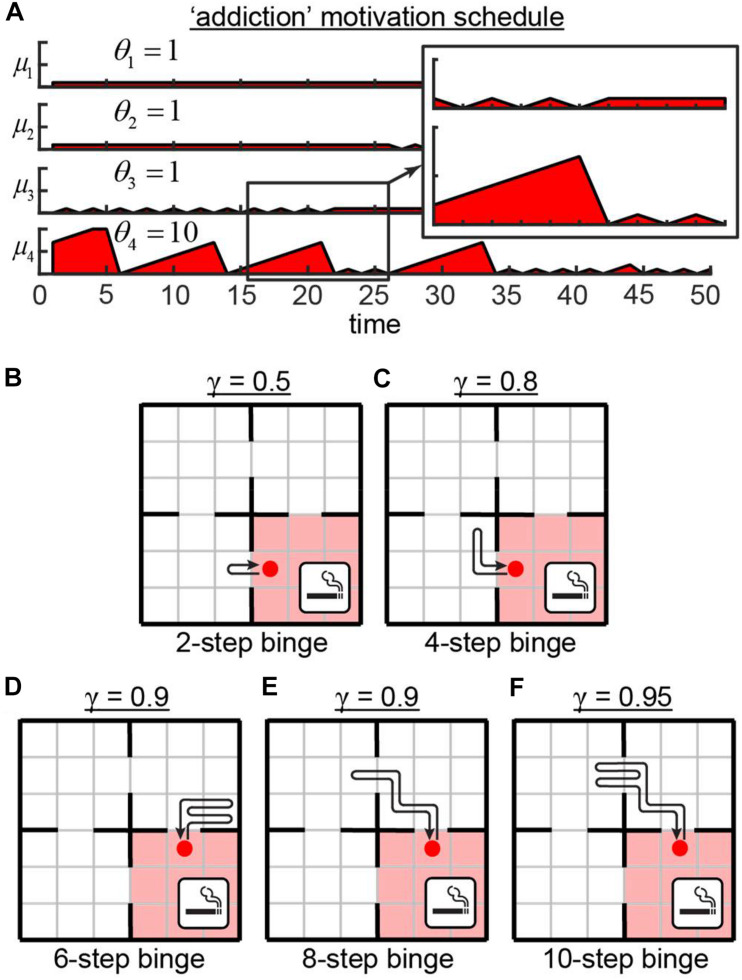
Addiction model. **(A)** Motivation schedule for model of addiction. In the first three rooms, motivations are allowed to grow up to the value of **θ = 1**. In the fourth (“smoking”) room, the motivation may grow to the value of **θ = 10**. **(B–F)** Strategies learned by the network for various values of the discounting factor **γ**, defining the relative values of the future rewards. Intermediate value of **γ = 0.9** yields different behaviors **(D,E)**.

We then tested the behavior of our network in the Four Demands task in which one of the rooms and its associated motivation is different from others. We show below that agents controlled by such a network learns to displays some elements of addictive behavior. Smoking cessation leads to a short-term increase in the number of cravings per day, followed by a longer-term reduction in craving rate (O’Connell et al., 1998). To mimic this feature and to model the nicotine addiction in our network, we changed the schedule of motivation dynamics in one of the rooms (Figure 3A). In this example, in the first three rooms, the motivation values were limited by θ_1..3_ = 1. In the fourth room – the “smoking” room – the motivation could increase to its maximum value of θ_4_ = 10. We did not include the gradual reduction in the rate of cravings due to smoking “cessation” into our model for simplicity. After training, the agent spent most of the time in the close proximity of the “smoking” room periodically entering it to receive a relatively high level of reward (Figures 3B–F). Overall, the behavior of the agent depended on the value of the discounting factor γ defining the relative values of future rewards. In cases of the small γ, the agent did not wait until its motivation for “smoking” reached its maximum value of μ_4_ = 10, and immediately reentered the “smoking” room after leaving it. This behavior can be explained by the fact that corresponding small values of the discounting factor γ = 0.5 devalued the rewards in distant future, therefore making it unreasonable for the agent to wait for high values of μ_*4*_. For larger γ, the agent performed longer excursions outside the ‘smoking’ room. The resulting variability of the binging strategies (Figure 3) goes in line with individual differences in craving rates and behaviors reported in smoking addiction literature (Ikard et al., 1969; McKennell, 1970; Shiffman, 1993). Overall, the networks with motivational salience can display a variety of behavioral features for different motivation dynamics, such as addiction. These features emerge from varying the dynamics of motivation as a function of time. We suggest that RL models of motivation, such as the one described above, may be useful in modeling real-world behaviors, including potential explanations for the mechanisms of addiction.

### Transport Network

**FIGURE 4:**
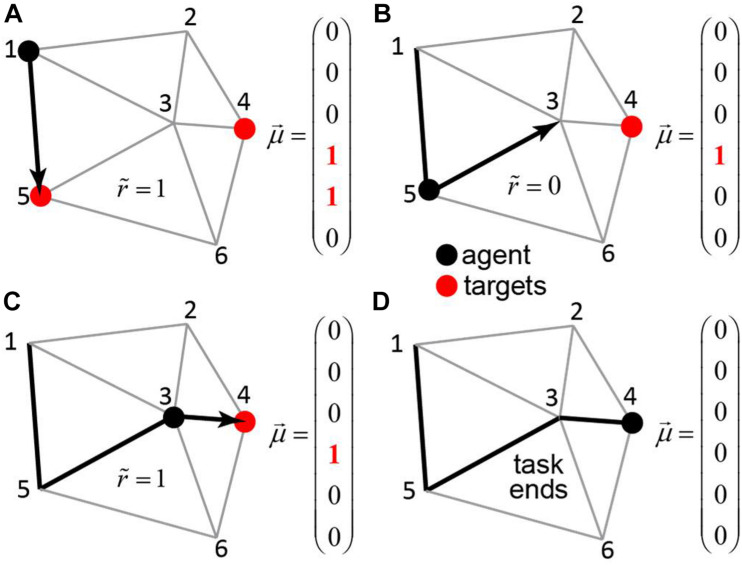
The transport network. An agent (black dot) navigates in a network of roads connecting the cities – each associated with its own binary motivation. The subjective reward value is equal to the value of the motivation vector $\vec{\mu }$ at the position of the agent, less the distance traveled. When the agent visits a city with non-zero motivation (red circle), the motivation toward this city is reset to zero. The task continues until $\vec{\mu }=0$. **(A–D)** The steps of the agent through the network (black arrows), the corresponding motivation vectors, and subjective reward values.

Our next goal is to present an example of a somewhat more complex task, in which motivational salience can help formulate hierarchical behavior. We now consider an example of an agent navigating in a system of roads connecting N cities (Figures 4A–D). The goal of the agent is to visit a certain subset of the cities, referred to as targets. The visiting order is not important, but the agent is supposed to utilize the route of minimal length. This problem is similar to the vehicle routing problem (Dantzig and Ramser, 1959), although we do not require the agent to return to the city of origin for simplicity.

**FIGURE 5:**
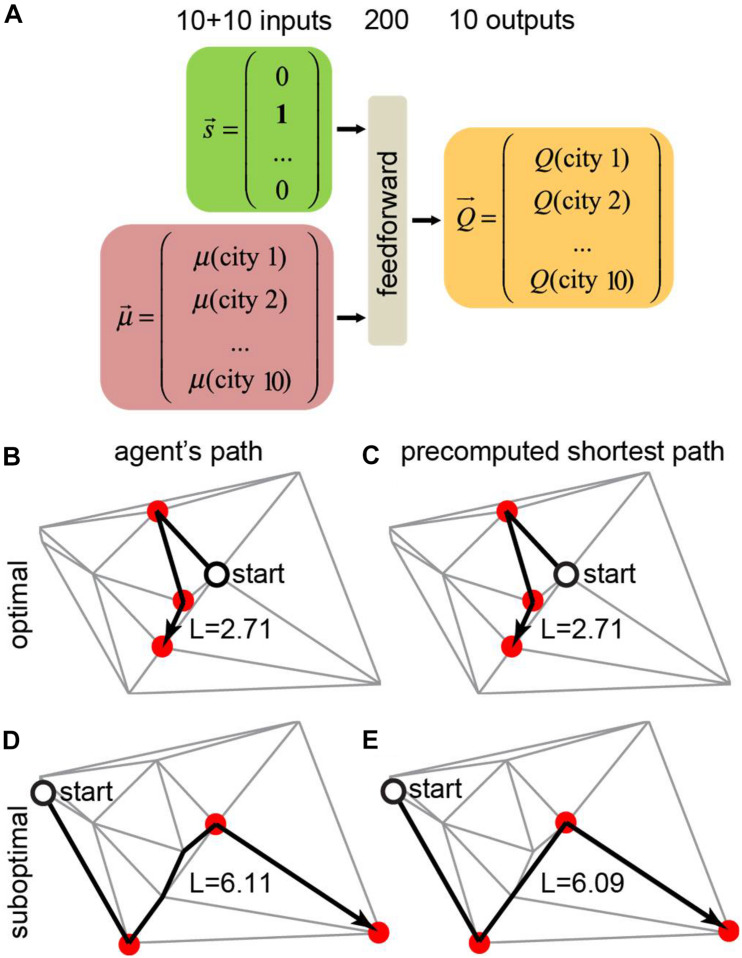
Training a neural network to find the shortest route to visit a subset of target cities using the motivation framework. **(A)** In this example, the total number of cities is *N* = 10, and the number of target cities to visit is *m* = 3. The neural network receives the agent’s position and motivation vectors as inputs and computes the *Q*-values for all available actions. **(B,C)** The trained agent **(B)** took the correct shortest path solution **(C)**. This scenario accounts for 82% of the test examples. **(D,E)** The trained agent **(D)** took a different route than the shortest path solution **(E)**. This scenario accounts for 18% of the test examples.

**FIGURE 6:**
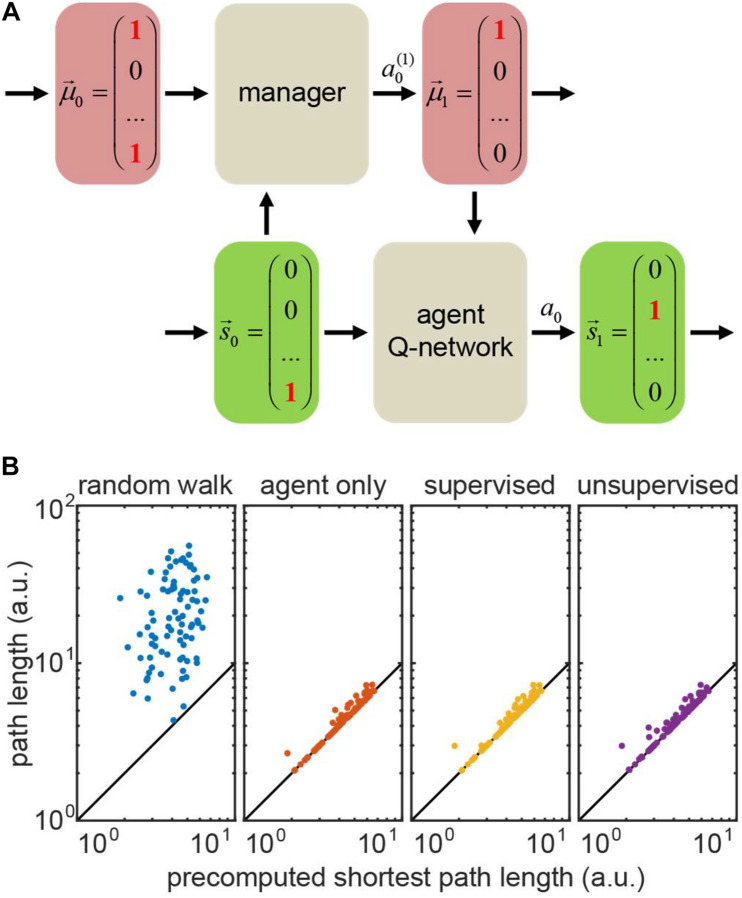
**(A)** Hierarchical reinforcement learning (HRL) setting for the transport network example. Bottom row: the agent Q-network receives current state (position) and motivation, and then computes the *Q*-values for transitioning to the other states (positions). Top row: the manager that makes changes in motivation. The manager can be represented by a hardcoded set of rules (random walk/agent-only simulation), or a Q-network (supervised/unsupervised simulations). **(B)** Performance of four representative models on 100 test runs trained in the same network: actual path lengths versus precomputed shortest path solutions. In the agent only simulation, the agent is supplied with the accurate motivation, whereas in hierarchical models (supervised/unsupervised), the motivation vector is computed by the manager network. The diagonal: identity line.

We trained a neural network that receives the agent’s state (position) and the motivation vector as inputs and computes the Q-values for all available actions (connected cities) for the given position (Figure 5A). In every city, the agent receives a reward equal to the value of the motivation vector at the position of the agent. The network is also punished at every link between cities in proportion to the length of this link. To model a real-life agent that balances reward maximization and policy complexity (Parush et al., 2011), we approximated the *Q*-values using a simple one-hidden-layer neural network, and selected actions using softmax policy (Parush et al., 2011) over these *Q*-values. We trained the agent’s network using TD method by backpropagating reward prediction error (δ) signal. Trained neural networks produced behaviors that closely match the shortest path solution (Figure 5B). Examples of the trained agent behaviors are shown in Figures 5B–E. In 82% of the test examples, the agent traveled the shortest path (Figures 5B,C). In the remaining 18% of cases, the path lengths of the trained agents were close to the shortest path solution (Figures 5D,E, 6B). The path traveled by an agent exceeded the shortest path only by 3% on average. Overall, our findings suggest that networks with motivation can solve fairly complex navigation problems. In doing so, the agent is not provided with any particular target, but, instead, learns to select the next target based on the balance between attractiveness of different goals measured by the closeness to the shortest path solution.

### Motivational Salience Can Help Implement Hierarchical Behavior

Here, we propose that motivation may present a mechanism of how hierarchical reinforcement learning (HRL) algorithms can be implemented in the brain. As described above, actions in the motivation-based RL are chosen on the basis of Q-function *Q*(*s*_*t*_,*a*_*t*_,μ_*t*_). An action *a*_*t*_ chosen at certain time point usually maximizes the Q-function, representing the total expected future reward, and leads to the transition of the agent to the new state: *s*_*t*_→*a*_*t*_→*s*_*t* + 1_. Because of the dependence of the Q-function on motivation, chosen action depends on the variable μ representing motivational salience. We argued above that motivation allows RL to have the flexibility of a rapid change in behavioral policy when the need of an animal fluctuates. The same mechanism can be used to implement HRL, if motivation μ is supplied by another, higher-level ‘manager’ network with its own Q-function, $Q^{\left(1\right)}\,\left(\mu_{t},a_{t}^{\left(1\right)},\mu_{t}^{\left(1\right)}\right)$. When the higher-level network picks an action $a_{t}^{\left(1\right)}$, it leads to a change in the motivational state for the lower-level network: $\mu_{t}\to a_{t}^{\left(1\right)}\to \mu_{t+1}$ thus rapidly changing the behavior of the latter. The ‘manager’ network could on its own be controlled by a higher-level manager via its own motivation $\mu_{t}^{\left(1\right)}$. The decision hierarchy could be very complex, if it includes several management levels, with the dynamics of motivation on level *l* determined via Q-function computed on level *l+1*: $Q^{\left(l+1\right)}\,\left(\mu_{t}^{\left(l\right)},a_{t}^{\left(l+1\right)},\mu_{t}^{\left(l+1\right)}\right)$ and $\mu_{t}^{\left(l\right)}\to a_{t}^{\left(l+1\right)}\to \mu_{t+1}^{\left(l\right)}$.

To demonstrate a hierarchical system in out transport network example, we implemented a *manager* network which received the agent’s state (position) and the estimate of the prior motivation vector as inputs. The manager network computed the *Q*-values for all possible *manager’s* actions $a_{t}^{\left(1\right)}$. These actions yielded changes in estimated motivation for the next time step, i.e., $\mu_{t}\to a_{t}^{\left(1\right)}\to \mu_{t+1}$ (Figure 6A). The manager supplied the estimated motivation for the next time step μ_*t+1*_ to the trained agent, a Q-network which used μ_*t+1*_ alongside with its own true state to transition to a new state, *s*_*t*_→*a*_*t*_→*s*_*t* + 1_, and collect a corresponding reward. We trained the manager network using the TD method. We considered two types of the manager reward schedules, as described below.

To model the tasks with full knowledge of the agent’s motivation – where one only needs to infer the rules of motivation dynamics – we first implemented the *supervised* learning model for the manager network. The training set consisted of prerecorded pairs of true motivations before and after the agent’s action. We provided the manager network with a positive reward for a *manager’s* action that changed the motivation correctly, or with a matching negative reward otherwise. For an incorrectly taking action of *doing nothing*, we assigned only a half of the usual negative reward – to discourage erroneous changes of estimated motivation. We matched the positive and negative rewards for the manager to prevent it from reinforcing erroneous actions (e.g., setting to zero a motivation component which is already zero), or being overcautious (taking only *do nothing* actions). Trained using this method, the *supervised* manager learns the agent’s motivation dynamics correctly, causing no deficiency in its performance (exceeding the shortest path by 3% on average – same as the agent supplied with correct motivation; Figure 6B, compare “supervised” to “actor only”).

We then implemented the *unsupervised* RL model for the manager. In this case, the manager is not supplied with the correct motivation vector at each step of training, and, instead, has to infer motivation dynamics for the transport network using agent’s rewards only, in a true RL fashion. The training occurred through interaction between the agent and the manager. At every step, the manager took an action to update the estimated agent’s motivation and communicated it to the agent. The agent then transitioned to a new state and collected a corresponding reward. We only propagated the *sign* of the agent’s reward to the manager, as the exact intrinsic reward of the manager may not be the same as for the actor. Similarly to the supervised case, we balanced positive and negative rewards for the manager (positive reward was larger than negative due to fewer occurrences). When a negative reward followed the manager’s action of *doing nothing*, we assigned the manager a half of the usual negative reward. We performed training in batches to average out diverse exploratory behaviors of the agents outside familiar settings (Henderson et al., 1982). We then evaluated the manager through the agent’s performance, as we assumed no knowledge of the agent’s true motivation. Figure 6B shows that the *unsupervised* manager learns the agent’s motivation dynamics, causing only a slight deficiency in its performance (exceeding the shortest path by 4% on average; Figure 6B, “unsupervised”).

Overall, the results above suggest that HRL networks with motivation may provide a powerful tool to infer motivation dynamics of the real-life agents. It is also possible that HRL mechanisms in the brain may have evolved from circuits implementing motivational salience. As such, HRL networks with motivation may be useful in interpretation of experimental data.

### Responses of Neurons in Ventral Pallidum (VP)

**FIGURE 7:**
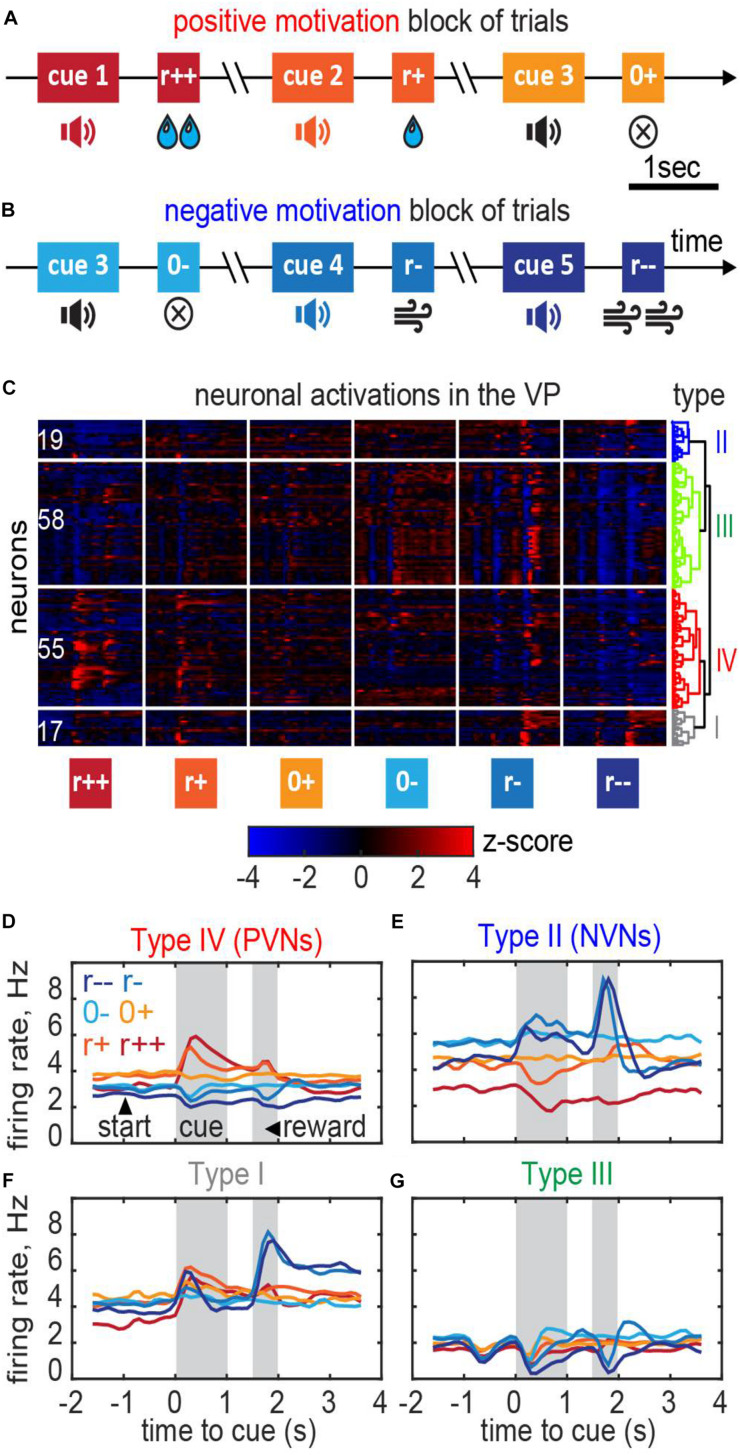
*Ventral pallidum* responses in the classical conditioning task with motivation. **(A,B)** A behavioral task for the recording. Trials were separated into blocks during which only rewards (water drops) or only punishment (air puffs) were delivered thus providing positive or negative motivation. **(C)** Responses of the VP neurons recorded in 3 mice clustered into the negative motivation neurons, positive motivation neurons, and neurons of mixed sensitivity. Dendrogram shows hierarchical clustering (see section “Materials and Methods”). **(D–G)** Average firing rates of the neurons in each cluster [cell type names follow Stephenson-Jones et al. (2020)]. **(D)** Positive valency neurons (PVNs) elevate their firing rate in reward condition in proportion with the reward magnitude; the baseline firing rate in PVNs in reward conditions is higher than in punishment conditions. **(E)** Negative valency neurons (NVNs) show the opposite trend. **(F,G)** Mixed sensitivity neurons (type I and type III) do not distinguish reward and punishment conditions.

To verify consistency of our theory with neuronal recordings we applied our model to data collected within a classical conditioning task with motivation (Stephenson-Jones et al., 2020). Below we provide a brief recap of the task; more details are described in Stephenson-Jones et al. (2020). Mice were trained to associate specific cues (sound tones) with different outcomes (Figures 7A,B). Specifically, the animals received one of five possible outcomes: a strong or weak reward (5 μl/1 μl of water); a strong or weak punishment (500 ms/100 ms of air puff); or nothing at all (0 μl of water/0 ms of air puff). Trials containing rewards/punishment were separated into different blocks to place the animals into positively or negatively motivated states. Trials with no reward/no punishment (in reward/punishment blocks, respectively) were identical and were indicated to the animals with the same sound tone (cue). In these trials, differences in behavioral or neuronal responses were unrelated to the reward magnitude, and solely relied on the animal’s motivation (motivated/demotivated). To study the underlying neuronal circuits, we recorded the activity of neurons in the VP, a brain area considered central to computing motivation (Berridge and Schulkin, 1989), in three mice while they were performing this task (Figures 7A,B). Overall, we obtained 149 well-isolated single neurons [Figure 7C; see (Stephenson-Jones et al., 2020) for details].

We classified these neurons based on their firing patterns type using an unsupervised clustering approach (“Materials and Methods”; Stephenson-Jones et al., 2020). We found that the neuronal population contained at least four functionally distinct types (Figure 7C). In the *positive valency* neurons (type IV or PVNs; 55 out of 149 cells), baseline neuronal activations increased during the expectation of rewards and decreased during the expectation of punishment (Figures 7C,D). In the *negative valency* neurons (type II or NVNs; 19 out of 149 cells), the activations followed the opposite rule (Figures 7C,E). In the *mixed sensitivity* neurons (type I and type III; 75 out of 149 cells), the responses were of the same sign in reward and punishment conditions (Figures 7C,F,G). Type names were assigned in accordance with (Stephenson-Jones et al., 2020).

The responses of VP neurons appeared to reflect the reward signs and amplitudes (Figures 7C–G), similarly to habenula-projecting globus pallidus (GPh) neurons (Stephenson-Jones et al., 2016) or dopamine neurons (Hong and Hikosaka, 2008). Unlike the GPh neurons, the responses of VP neurons also showed the following features:

(1) The responses of some of the VP neurons (in particular the responses of the PVN type cells) to the sound cue were more sustained than those of dopamine or GPh neurons (Cohen et al., 2012; Stephenson-Jones et al., 2016) and often persisted until the reward delivery (Figures 7C,D);
(2) The responses of the VP neurons to the reward expectation were modulated by the licking rate indicating coupling to motivation (Stephenson-Jones et al., 2020). By contrast, GPh neuron responses to expectation are stable over time (Cohen et al., 2012).
(3) The baseline firing rates of the VP neurons changed as mice transitioned between rewards/punishment blocks of trials (Figures 7C–E), potentially reflecting changes in motivational state. In particular, the baseline firing rates of the VP neurons in the identical no reward/no punishment trials were different (Figures 7C–E). GPh neurons do not tend to show such modulation (Stephenson-Jones et al., 2016).

Overall, our data suggests that the VP contains two populations of oppositely-tuned neurons, responding to rewards (Figure 7D) and punishment (Figure 7E). These neurons encode not only the reward values, but also motivational states. Further data and considerations supporting the conclusion that the VP neurons in our recordings are specifically tuned to motivation can be found in Stephenson-Jones et al. (2020).

**FIGURE 8:**
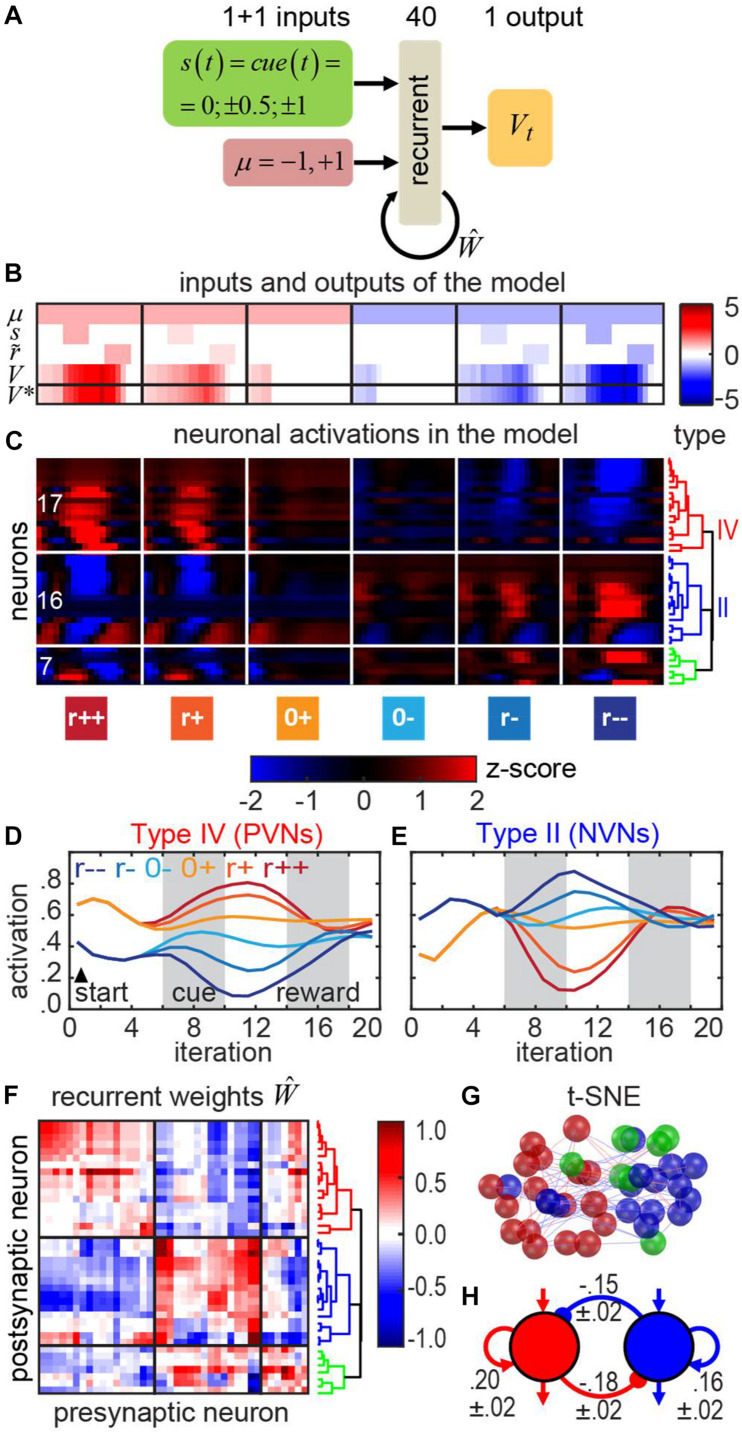
Recurrent neural network with motivation in the classical conditioning task. **(A)** The architecture of the RNN computing the V-function in this task. **(B)** Inputs and outputs of the RNN for each trial type. Inputs: motivation **μ**, cue ***s***, subjective reward value **$\tilde{r}$**. Outputs: V-function. Bottom row: the precomputed correct V-function ***V*^∗^**. Trial types (left to right): strong reward, weak reward, no reward, no punishment, weak punishment, strong punishment. **(C)** Responses of neurons in the RNN can be clustered into the positive motivation (red), negative motivation (blue), and neurons of mixed sensitivity (green). Dendrogram shows hierarchical clustering (see section “Materials and Methods”). **(D,E)** Average activities of the neurons in red and blue clusters in the model resemble those of PVNs and NVNs recorded in the VP. **(F)** Recurrent connectivity matrix. **(G)** t-SNE embedding of the RNN neurons based on their weights (spatial arrangement) corresponds to their clustering by the activity (red/blue/green color as in the panels **C,F**). **(H)** The push-pull circuit – a schematic representation of the recurrent connectivity in the model (annotated with the mean weights and the corresponding standard errors of mean; SEMs).

To see whether our model can reproduce these findings, we investigated artificial neural networks with motivation that were subjected to similar training procedure. Because the Pavlovian conditioning task includes time as variable (Figure 7), we chose to use recurrent neural network (RNN) as a basis of our model (Sutton and Barto, 1987; see section “Materials and Methods”). The network received two time-dependent inputs. One input described the cue (Figures 8A,B) – which we view here as a state of the environment because it indicates to the model what kind of trial it is currently in (weak/strong reward/punishment trial, or no reward/no punishment trial). Another input described motivation, which was constant within the entire trial. Motivation input was introduced to indicate to the model whether it is in a reward (μ = + 1) or punishment (μ = −1) block of trials. Motivation also allowed the model to distinguish the otherwise identical no reward/no punishment trials. We trained the network using backpropagation (see section “Materials and Methods”). The resulting inputs and outputs after training for various conditions are shown in Figure 8B.

We show (Figure 8B) that the network has learned a rational expectation of trial outcome as a function of time. For example, in the positive motivation trials (μ = + 1), early in the trial, before a cue is presented, the expected value of future reward *V*_*t*_(*s*_*t*_, μ_*t*_) starts from a low positive value, in expectation of future reward. As the cue arrives, the expected value of future reward *V*_*t*_ reasonably represents the expected outcome. For example, in the trials with the strong reward (the leftmost column in Figure 8B), the network adjusts its expectation to higher value after the cue arrives. For the trials with the weak reward (second column), no adjustment is necessary, and, therefore, reward expectation *V*_*t*_ remains unaffected by the cue. *V*_*t*_ increases slightly after the cue arrives due to the temporal discount γ = 0.9. For no reward trials (third column in Figure 8B), in the positive motivation case, the expected reward decreases after the cue arrives, due to the pessimistic prognosis predicted by the cue. In negative motivation cases (μ = −1, Figure 8B, columns 4–6), the behavior of the network is similar except for the sign. Overall, the network produces reward expectations *V*_*t*_ that accurately reflect motivation and future rewards.

We then examined the responses of individual neurons in the model. We clustered the population of artificial neurons using the same clustering approach as for the VP recordings (“Materials and Methods”; Stephenson-Jones et al., 2020). We found that neural population contained two groups of oppositely tuned cells (Figure 8C), similarly to the experimental observations in the VP (Figure 7C). These two clusters of neurons were elevating their baseline activity in reward (Figures 8C,D) and punishment (Figures 8C,E) trials, respectively. Here, we did not train the first, feedforward layer of the weights in our network model (between the inputs and the recurrent layer) leaving it initialized with random numbers in accordance with the Xavier rule (see section “Materials and Methods”). Although the results were qualitatively similar in the model where all layers are trained (i.e., we observed tuning of baseline activity to motivation, and of phasic activity to the expected reward magnitude), using the random first-layer weights resulted in relatively small motivation-related variation of baseline activity (Figures 8C–E), which was closer to what we observed in our VP data (Figures 7C–E).

We then examined the recurrent connectivity between these two groups of neurons in the model (Figure 8F). We found that similarly tuned neurons, i.e., cells belonging to the same functional cluster, tended to excite each other, whereas oppositely tuned cells tended to inhibit each other. Clustering of the neurons by the weights (spatial arrangement on Figure 8G) agreed with clustering by the activations (colors on the Figure 8F) (see section “Materials and Methods”) suggesting that this RNN circuit can be implemented with two distinct cell types.

Our model suggests the rationale for the existence of two oppositely tuned cell types. In the classical conditioning task, the conditioned stimulus (CS) and reward/punishment are separated by a temporal delay. During the delay, the network is expected to maintain a memory of upcoming punishment or reward, and, thus, acts as a working memory network (Her et al., 2016) which retains reward expectation in its persistent activity. This persistent activity is seen in both the responses of individual neurons in VP (Figures 7D,E) and the RNN neurons (Figures 8D,E). Persistent activity in RNN belongs to the class of parametric persistent responses often studied in working memory and decision-making tasks (Cannon et al., 1983; Goldman et al., 2003; Machens et al., 2005; Her et al., 2016). Previous studies of working memory and decision-making tasks (Cannon et al., 1983; Machens et al., 2005; Wong et al., 2007) suggest that persistent activity can be maintained by two groups of oppositely tuned neurons in a network architecture called the “push-pull” circuit. This solution is also discovered by our model (Figures 8F–H). In this type of circuit, memory is maintained via positive feedback. The positive feedback is produced by two forms of connectivity. First, similarly tuned neurons excite each other (Figure 8). Second, oppositely tuned neurons inhibit each other, which introduces effective self-excitation via disinhibition (Figure 8). Overall, our artificial RNN yields a prediction for the structure of connectivity implementing reward prediction in the classical conditioning setting.

Our RNN model presented above was obtained *ab initio* to represent the entire reward system. Our previous results suggest a lack of recurrent connectivity in the VP (Stephenson-Jones et al., 2020). We suggest that push-pull circuit in our model corresponds to the neurons upstream of the VP, e.g., to the prefrontal cortex (PFC) known to maintain the cue-reward associations (Gottfried et al., 2003; Bray and O’Doherty, 2007). The VP neurons, maintaining positive or negative tuning to motivation, may therefore correspond to the final feedforward layer of our model (Figure 8A).

To examine whether our model with motivation predicts the VP neural signals better than the model that does not incorporate motivation, we additionally trained networks with non-informative constant input (μ^∗^ = 0≠μ) instead of varying motivational input. The subjective values of the rewards in the task did not change ($\tilde{r}=\mu \,r\neq \mu^{*}\,r$). Such networks learned to contain two neuronal types – tuned positively and negatively to the rewards. Activations of these neurons, however, did not show tuning of their baseline activations to reward/punishment blocks of trials observed in the VP. Non-motivated agents were therefore inconsistent with the experimental observations. In addition, non-motivated models could not distinguish the neutral stimuli (no reward/punishment, the same CS) presented during positive and negative motivation blocks of trials. Although the number of cell types learned by motivated and non-motivated agents matched, only the activation patterns of cells in motivated agent resembled those recorded in the brain.

## Discussion

Motivational salience, which we call here, for brevity, motivation, has been defined previously as the need-based modulation of reward magnitude (Zhang et al., 2009; Berridge, 2012). Here we proposed an RL approach to the neural networks that can be trained to include motivation into the calculation of action. We considered a diverse set of example networks that can solve different problems using a similar architecture. In each task, we aimed to use a simple model capable of successfully learning the task. This approach both minimized the training time for each model, and constrained the models to generalize their behaviors across the inputs instead of memorizing the input-output pairs. The networks received both current motivation and state variables as inputs and were trained to compute the magnitude of cumulative motivation-dependent future rewards (Q-function). The action was then selected as a maximum over the Q-function. The network weights were updated using TD rule via the conventional backpropagation algorithm. We found that the networks can learn correct behaviors in this setting, including behaviors that reflect relatively complex scenarios of future motivation changes. Thus, our model, in the transport network example, is capable of solving an NP-complete task without relearning the connection weights.

Our approach is based on the previous model by Zhang et al. (2009), with a few critical differences. First, in the aforementioned work the state of the agent (reflecting the cue/conditioned stimulus, CS) was the only input of a value function; we considered the value function to be explicitly a function of the state and motivation. This way, our models were able to learn the relation between the state, motivation, and their joint incentive value. Second, to interpolate between the multidimensional inputs of the value function, we used deep neural networks. Deep RL models are capable of learning the generalized rules in their weights from the first principles. In our case, the models were capable of generalizing the relation between the rewards, motivations, and their incentive values (i.e., the product of the reward and motivation) and were also able to extrapolate their policies to the novel motivation schedules via developing new courses of action (e.g., the “delayed migration” policy). Third, in our models, the motivations were multidimensional and dynamic, forcing the agents to learn the dynamics of motivations to develop the optimal behaviors. Overall, our work combines the Berridge’s model of motivation with deep RL and previous models of motivational drives to provide an interpretable framework for studying motivated behaviors and their algorithmic rationale in real-world agents and settings.

Although motivation seemingly can be viewed as a part of the agent’s state, there are multiple reasons to consider them separately. First, motivation is generally a slowly changing variable. Thus, an animal’s appetite does not change substantially during a few seconds of a single behavioral trial. At the same time, the animal’s actions may lead to immediate changes of its position. Second, the research in neuroscience suggests that motivation and state may be represented and computed separately in mammalian brain. Whereas motivation is usually attributed to the regions of the reward system, such as the ventral pallidum (VP) (Berridge and Schulkin, 1989; Berridge, 2012), the state is likely to be computed elsewhere, e.g., in the hippocampus (Eichenbaum et al., 1999) or cortex. Such distinction in the brain may be based on an algorithmic rationale that facilitates computations and is yet to be understood. Finally, in hierarchical RL (HRL) implementation, motivation is provided by a higher-level network, while information about the state is generated externally. For these reasons, in this work we consider an agent’s state ${\vec{s}}_{t}$ and its motivation $\vec{\mu }$ separately.

Although the Q-function with motivation (2) is similar to the Q-function in goal-conditioned RL (Schaul et al., 2015; Andrychowicz et al., 2017), the underlying learning dynamics of these two models are different. Motivated behavior simultaneously pursues multiple distributed sources of dynamic rewards. The Q-function therefore accounts for the internal motivation dynamics. This way, an agent with motivation chooses what reward to pursue – making it different from RL with subgoals (Sutton et al., 1999). As we show in this work, simple motivational schedules give rise to large varieties of behaviors. A reduction in numbers of handcrafted features suggests that motivation could provide a step toward more general methods of computation – a goal identified recently by Richard Sutton (Sutton, 2019).

Our model of motivation is consistent with the large body of existing motivational models and behavioral observations. In a recent work, Keramati and Gutkin (2014) show that *homeostatic RL* explains prominent motivation-related behavioral phenomena including anticipatory responding (Mansfield and Cunningham, 1980), dose-dependent reinforcement (Hodos, 1961), potentiating effect of deprivation (Hodos, 1961), inhibitory effect of irrelevant drives (Dickinson and Balleine, 2002), etc. Although homeostatic RL defines the rewards as the gradients of the cost function with a fixed point, the theoretical predictions generalize to the models with linear, or approximately linear, multiplicative motivation. We therefore expect the behaviors of our models to be consistent with the large body of experimental data described above (Hodos, 1961; Mansfield and Cunningham, 1980; Dickinson and Balleine, 2002).

Biologically-grounded choices of motivation dynamics enable our model to reproduce realistic behaviors, including those related to drug addiction. Here we show that a simplistic model, where motivation toward “smoking” grows large compared to motivations toward the other rewards, qualitatively accounts for the binging behavior. Our model suggests that the smoking frequency can be explained with the temporal discounting parameter γ defining the relative impact of the rewards near and far in the future. Our framework, offering a way to derive behaviors from the first principles, can be combined with the classical results regressing the craving rates to a variety of environmental cues (e.g., McKennell, 1970) to build finetuned models of addicted behaviors. For example, motivational dynamics may change over time. Addictive drugs can become less rewarding (‘liked’) after repeated experience despite increases in the motivational salience and/or craving for drugs – which can be accounted for in the model with an additional layer in motivation hierarchy (akin to the Transport network task). The discrepancy between low ‘liking’ of the drug and the high rate of cravings can be formalized as the difference between motivational salience and motivational vigor, the generalized willingness to expend energy toward a reward. Motivational vigor can be incorporated into the model by the means of the actor-critic formalism (Sutton and Barto, 1998) which computes log likelihoods for every action. Including these and other parameters to our motivation framework may help building detailed models of addictive behaviors in future work.

We trained recurrent neural networks (RNNs) to estimate future motivation-dependent subjective values of the reward in the Pavlovian conditioning task. In contrast to purely feedforward networks, the RNNs allow learning the temporal sequences of events such as the associations between reward-predicting cues (conditioned stimuli, CS) and following rewards (unconditioned stimuli, US). The ability to learn the temporal US-CS associations makes RNNs a rational choice for the models of animal behavior and neuronal activity in Pavlovian conditioning tasks (Sutton and Barto, 1987). Since the structure of network for computing motivation-dependent reward expectations is not fully understood, modeling this circuit as an RNN seems to be a simple and plausible first step, similarly to the models of persistent activity and working memory. It is not clear at the moment whether RNN obtained here is fully contained in VP or is represented by some other part of the reward circuit. Our mathematical model does not specify where the recurrent connectivity facilitating the persistent activity is formed; such structure could occur in PFC where neurons are known to maintain cue-reward associations (Gottfried et al., 2003; Bray and O’Doherty, 2007), in VP, or in some other brain region. Previously published findings suggest that VP may not contain connectivity that is strong enough to maintain persistent activity (Stephenson-Jones et al., 2020). Our study may motivate the search for the recurrent circuit that can maintain cue-reward associations.

We found that neurons in the RNNs trained to recognize motivation can be clustered into two oppositely tuned populations: positive and negative motivation neurons. These populations display increased firing in reward and punishment trials, respectively. Similar two groups of neurons are found in the previously published data (Stephenson-Jones et al., 2020) on neural responses in the mouse’s VP: a basal ganglia region implicated in motivation-dependent estimates of reward (Richard et al., 2016). Thus, our neural networks develop response patterns comparable to experimentally observed in the brain.

We found that the recurrent network structure in this Pavlovian conditioning case is compatible with the conventional models of working memory. The general idea is that the information about an upcoming reward – once supplied by a cue – is maintained in the network due to the positive recurrent feedback. This feedback can be produced by disinhibition between two oppositely tuned populations of neurons, namely positive and negative motivation sensitive cells. Thus, the presence of two subpopulations of neurons may be a consequence of the functional requirements on the network to maintain persistent variables within a trial. This function is reflected in both neural responses and architecture.

Motivation offers a framework compatible with other methods in machine learning, such as R-learning, goal-conditioned RL, and hierarchical RL (HRL). *R-learning* is an average-reward reinforcement learning model (Schwartz, 1993; Sinakevitch et al., 2018). Specifically, the cumulative sum of future rewards is computed with respect to the average level of reward. The average reward level is computed across multiple trials, which makes it similar to motivation. In *goal-conditioned RL* – the closest counterpart to RL with motivation – the Q-function depends on three parameters: $Q\,\left({\vec{s}}_{t},a_{t},g\right)$, where *g* is the current static goal. In the motivation framework, multiple dynamic goals are present at the same time and it is up to an agent to decide which one to pursue – based on the future motivation dynamic learned by the network. *HRL methods* include the options framework (Sutton and Barto, 1987; Sutton and Barto, 1998), RL with subgoals (Sutton et al., 1999), feudal RL (Dayan and Hinton, 2000; Bacon and Precup, 2018), and others. In HRL, complex tasks are solved by breaking them into smaller, more manageable pieces. In both the case of motivated agents and HRL, the reward function is manipulated by an external process, such as a higher level manager (Sutton et al., 1999). HRL approaches have several advantages compared to traditional RL, such as transfer of knowledge from already learned tasks and the ability to faster learn solutions to complex tasks. Although HRL methods are computationally efficient and generate behaviors separated into multiple levels of organization – which resemble animals’ behavior – a mapping of HRL methods to brain networks is missing. Here, we suggest that motivation offers a way for HRL algorithms to be implemented in the brain. In case of motivation, the goal of the agent is not explicitly specified and may shift in course of behavior if motivational variables change their values. Moreover, multiple goals may simultaneously be presented to an agent, whose aim is to select the one that yields the highest subjective reward. We present an example of how HRL can be implemented in motivation setting for the case of transport network.

Overall, we suggest that motivation-based networks can generate complex ongoing behaviors that can rapidly adapt to dynamic changes in an organism’s demands without changes in synaptic strengths. Thus, neural networks with motivation can both encompass more complex behaviors than networks with a fixed reward function and be mapped onto neuronal circuits that control rewarded behaviors. Since animal performance in realistic conditions depends on the states of satiety, wakefulness, etc., our approach should help build more realistic computational models that include these variables.

## Materials and Methods

### The Four Demands Task

To optimize the behaviors in the Four Demands task, we implemented a feedforward neural network as described below. On the input, the network received an agent’s state and motivation. The state variable contained an agent’s position, which was represented by a 36-dimensional one-hot vector. The motivation was represented by a 4-dimensional integer vector. From both state and motivation variables, we subtracted the mean values. To balance the contributions of state and motivation to the network, we normalized their variances to 1 and 9, respectively, since the ratio of the number of these variables is 4/36. The inputs of the network were propagated through three feedforward hidden layers (100 sigmoid units each), and a feedforward output layer (5 linear units) (Figure 2A). We trained the network to compute the *Q*-values of the possible actions: to move left, right, up, down, or to stay.

On every iteration, we picked an action, corresponding to the largest network output (*Q*-value). With probability ε, we replaced the selected action with a random action (ε -greedy policy; ε decreased exponentially from 0.5 to 0.05 throughout simulation; in case of random walk agents, we set ε = 1). If the selected action resulted in a step through a “wall,” the position remained unchanged; otherwise we updated the agent’s position. For the agent’s new position, we computed the subjective reward value ($\vec{r}\cdot \vec{\mu }$), and used Bellman equation (γ = 0.9) to compute TD error (Eq. 4). We then backpropagated the TD error through the network to update its weights [initialized using Xavier rule (Glorot and Bengio, 2010)]. Specifically, we used the network activations, corresponding to the previous step, and backpropagated TD-error only through the output, corresponding to the selected action. For each model, we performed 4⋅10^5^ training iterations with the learning rate decreasing exponentially from 3⋅10^−3^ to 3⋅10^−5^.

We trained the network using various motivation schedules as follows. Each reward component was set equal to one in a corresponding room, and to zero elsewhere (Figure 1B). Each component of the motivation was increased by one on every iteration (Figure 1C). If a component of motivation μ_*n*_ reached the threshold θ_*n*_, we stopped increasing this component any further. If the reward of a type *n* was consumed on current iteration, we dropped the corresponding component of motivation μ_*n*_ to zero. For motivated, non-motivated, and random walk agents, we trained 41 model each (123 models total) with motivation threshold θ_1_ = θ_2_ = θ_3_ = θ_4_ ranging from 1 to 100, spaced exponentially, one training run per unique θ value. To check whether a single network can perform optimally under various motivation schedules, we also trained a separate model with an additional input (θ) in minibatches of 10 iterations corresponding to θ = 1…10. To mimic addiction, we also trained a separate model with θ_1_ = θ_2_ = θ_3_ = 1, and θ_4_ = 10 (Figure 3A), using various values of γ = 0.5;0.8;0.9;0.95. For each run, we displayed sequences of agent’s locations to establish correspondence between policies and average reward rates.

### The Transport Network Task

To build an environment for the transport network task, we defined the locations for 10 “cities” by sampling *x*− and *y*− coordinates from the standard normal distribution *N*(0,1). For these locations, we computed Delaunay triangulation to define a network of the roads between the cities. For each road (Delaunay graph edge), we computed its length – the Euclidean distance between two cities it connects. We then selected multiple random subsets of 3 cities to be visited by an agent: the training set (10^5^ target subsets), and the testing set (100 different target subsets).

To navigate the transport network, we implemented a feedforward neural network as described below. On the input, the network received an agent’s state and motivation. The state variable contained an agent’s position, which was represented by a 10-dimensional one-hot vector. The motivation was represented by a 10-dimensional binary vector. To specify the agent’s targets, we initialized the motivation vector with 3 non-zero components μ_*i*_1__…_μ*i*_3__, corresponding to the target cities *i*_1_…*i*_3_. The inputs of the network were propagated through a hidden layer (200 Leaky ReLU units; leak α = 0.2), and an output layer (10 leaky ReLU units; leak α = 0.2). We trained the network to compute the *Q*-values of the potential actions (visiting each of the cities).

On every iteration within a task episode, we picked an action to go from the current city to one of the immediately connected cities, then we updated the current position. To choose the action, we used the softmax policy (β = 0.5) over the *Q*-values of the available moves. When the motivation μ_*j*_ toward the new position *j* was non-zero, we yielded the reward of 5, and dropped the motivation μ_*j*_ to 0. On every iteration, we reduced the reward by the distance traveled within this iteration. The task episode terminated when all the components of motivation were equal to zero. On every iteration, we used Bellman equation (γ = 0.9) to compute the TD error. We backpropagated the TD error through the network to update its weights (initialized using Xavier rule). For each model, we performed training on 10^5^ task episodes with the learning rate decreasing exponentially from 10^−2^ to 10^−4^. To assess the model performance, we evaluated the model (β = 10) on the testing set and compared the resulting path lengths one-by-one to the precomputed shortest path solutions.

To infer motivation dynamics from the trained agent, we implemented a manager neural network. On the input, the manager network received the agent’s state, and an estimate of the agent’s motivation (details below) in the same format as for the agent network. The inputs were propagated through three hidden layers (200 Leaky ReLU units; leak α = 0.2), and an output layer (11 leaky ReLU units; leak α = 0.2). We trained the network (using Bellman equation with γ = 0.9 and backpropagating the TD error) to compute the *Q*-values of all possible manager actions (set any component of the estimated motivation to zero, or do nothing). On every iteration, we picked an action, corresponding to the largest network output (*Q*-value). With probability ε, we replaced the selected action with a random action (ε-greedy policy; ε = 0.1).

In the supervised learning case, we trained the manager network (learning rate decreasing exponentially from 10^−3^ to 10^−6^) on 2⋅10^5^ prerecorded task episodes performed by the trained agent operating under correct motivation schedule (as described above) and initialized with random starting positions and targets. When the manager network predicted correct change of the agent’s motivation (supplied to the manager on the input), it received a reward of +2; otherwise a reward of −2. If a negative reward was received because of a “do nothing” manager action, we reduced it to −1.

In the unsupervised case, we trained the manager network (learning rate decreasing exponentially from 10^−2^ to 10^−5^, reduced to 10^−3^ whenever initially larger) using minibatches of 50 iterations on 2⋅10^5^ task episodes performed by the trained agent operating under manager-supplied motivation and initialized with random starting positions and targets. The estimated motivation, edited by the manager network on current step, served as an input to the manager network on the next step. The initial estimated motivation matched the agent’s initial targets. We manually terminated task episodes that lasted longer than 50 iterations. When the agent received a positive reward, we assigned the manager a reward of +5; otherwise a reward of −2. If a negative reward was received because of a “do nothing” manager action, we reduced it to −1.

We tested the manager networks (deterministic policy; ε = 0.0) maintaining its own estimate of the agent’s motivation and supplying it to the trained agent (softmax policy; β = 10), which operated on the same testing set as before. We compared the resulting path lengths one-by-one to the precomputed shortest path solutions.

### Pavlovian Conditioning Task

To build a circuit model of motivation in Pavlovian conditioning task, we replicated *in silico* the experiment where mice learned to associate sound cues with zero/weak/strong rewards/punishments, and reward/punishment trials were grouped in separate blocks to motivate/demotivate animals (Stephenson-Jones et al., 2020). We implemented a recurrent neural network, and trained it on sequences of 20 iterations (time steps) representing time within individual trials. For each individual trial, we first randomly chose whether the agent would be rewarded or, alternatively, punished (positive or negative motivation trials, respectively). We then randomly chose the strength of the upcoming reward/punishment to be zero, or weak, or strong. Depending on the type and strength of reward/punishment, we generated a cue (conditioned stimulus, CS): 0 for zero reward; 0.5 for weak reward; 1 for strong reward; 0 for zero punishment; −0.5 for weak punishment; −1 for strong punishment. The cues for zero reward and zero punishment were identical; thus, zero reward/zero punishment trials could be only distinguished based on motivation. We then passed motivation and cue to the network, as described below.

The cue input to the network was represented with the state variable. As the cue in Pavlovian conditioning task was only briefly presented shortly after the beginning of the trial (Stephenson-Jones et al., 2020), the state variable was initially equal to 0 (“silence,” time steps 1–5 out of 20), then equal to the CS as defined above (“sound tone,” time steps 6–10 out of 20), then again equal to 0 (“silence,” time steps 11–20 out of 20) (Figure 8B, the 2nd row). The motivation input to the network (1 for positive and −1 for negative) was constant throughout the entire trial (all 20 time steps; Figure 8B, the 1st row). The inputs to the network were propagated through a recurrent layer (40 sigmoid units), and a feedforward output layer (1 linear unit) (Figure 8A). We trained the network (recurrent and feedforward output layers) to compute the *V*-values (discounted sums of future expected subjective reward values) for each time step within the trial.

The subjective reward values (of unconditioned stimuli, US) were computed as a product of (positive) reward/punishment size and motivation – and thus numerically matched the CS values. As the rewards/punishments in Pavlovian conditioning task were delivered only shortly before the end of the trial (Stephenson-Jones et al., 2020), the subjective reward signal to the network was initially equal to zero (“no reward/no punishment yet,” time steps 1–13 of 20), then was equal to the US as described above (“reward/punishment,” time steps 14–18 out of 20), then again equal to 0 (“no more reward/no more punishment,” time steps 19–20 out of 20) (Figure 8B, the 3rd row). We used this subjective reward signal in Bellman equation (γ = 0.9) to compute a TD error for every time step (similarly to Eq. 4, without dependence on actions). We then backpropagated the TD errors through time to update the network’s weights [initially drawn from the uniform distribution *U*(−10^−5^,10^−5^)]. We did not update the weights between the inputs and recurrent layer [initialized using Xavier rule (Glorot and Bengio, 2010)]. For backpropagation, we used the network activations corresponding to the previous time step. We performed training on 3⋅10^5^ batches of 20 sequences each with the learning rate decaying exponentially from 10^−2^ to 10^−4^.

We clustered the neurons that we previously recorded in the VP, and the recurrent neurons in RNN after training, as follows (Stephenson-Jones et al., 2020). First, we computed the first three PCA components of the *z*-scored firing rates of all neurons in the strong reward and punishment trials. Then, we used these principal components in hierarchical clustering (Euclidean distance; complete agglomeration). To see whether the neurons within the same activity-based clusters in RNN have similar connectivity, we computed the weight-based correlation matrix between the recurrent neurons using the vectors of their postsynaptic weights. We then displayed the weight-based correlations in RNN using t-SNE (Maaten and Hinton, 2008) (*p=30*) and color-coded the neurons with respect to their activity-based clusters.

## Data Availability Statement

Requests to access these datasets should be directed to koulakov@cshl.edu.

## Author Contributions

All authors listed have made a substantial, direct and intellectual contribution to the work, and approved it for publication.

## Conflict of Interest

The authors declare that the research was conducted in the absence of any commercial or financial relationships that could be construed as a potential conflict of interest.
